# A novel emaravirus comprising five RNA segments is associated with ringspot disease in oak

**DOI:** 10.1007/s00705-021-04955-w

**Published:** 2021-01-18

**Authors:** Marius Rehanek, Susanne von Bargen, Martina Bandte, David G. Karlin, Carmen Büttner

**Affiliations:** 1 Division Phytomedicine, Albrecht Daniel Thaer-Institute of Agricultural and Horticultural Sciences, Humboldt-Universität zu Berlin, Lentzeallee 55/57, 14195 Berlin, Germany; 2 Independent Scholar, Marseille, France

## Abstract

We report the complete nucleotide sequence of the genome of a novel virus in ringspot-diseased common oak (*Quercus robur* L.). The newly identified pathogen is associated with leaf symptoms such as mottle, chlorotic spots and ringspots on diseased trees. High-throughput sequencing (HTS, Illumina RNASeq) was used to explore the virome of a ringspot-diseased oak that had chlorotic ringspots of suspected viral origin on leaves for several years. Bioinformatic analysis of the HTS dataset followed by RT-PCR enabled us to determine complete sequences of four RNA genome segments of a novel virus. These sequences showed high similarity to members of the genus *Emaravirus*, which includes segmented negative-stranded RNA viruses of economic importance. To verify the ends of each RNA, we conducted rapid amplification of cDNA ends (RACE). We identified an additional genome segment (RNA 5) by RT-PCR using a genus-specific primer (PDAP213) to the conserved 3´ and 5´termini in order to amplify full-length genome segments. RNA 5 encodes a 21-kDa protein that is homologous to the silencing suppressor P8 of High Plains wheat mosaic virus. The five viral RNAs were consistently detected by RT-PCR in ringspot-diseased oaks in Germany, Sweden, and Norway. We conclude that the virus represents a new member of the genus *Emaravirus* affecting oaks in Germany and in Scandinavia, and we propose the name “common oak ringspot-associated emaravirus” (CORaV).

**Supplementary Information:**

The online version contains supplementary material available at 10.1007/s00705-021-04955-w.

In Germany, chlorotic ringspots, mottling and chlorotic spots have been observed for decades on leaves of common oak (*Quercus robur* L.) [1]. Mechanical transmission of the symptoms to healthy oak seedlings failed, although graft transmissibility had been demonstrated earlier [2]. A viral cause for the observed symptoms was thought likely, and this hypothesis was supported by electron microscopy images of virus-like particles with spherical morphology [3]. Attempts to determine the causal agent by serological testing and molecular approaches excluded known viruses affecting *Quercus* [4].

Here, we report the complete genome sequence of a novel emaravirus based on its initial identification in a ringspot-diseased common oak tree in Kreuztal, Germany, using high-throughput sequencing (HTS, Illumina, HiSeq), first employing a random sequencing approach, followed by an emaravirus-enrichment strategy for library preparation [5]. In the HTS datasets, we identified sequence contigs relating to four genome segments of a putative novel emaravirus by using the Basic Local Alignment Tool X (BLASTx) against the NRPROT database, and we established diagnostic RT-PCR assays targeting RNA 2-RNA 4 of the novel virus [5]. The genus *Emaravirus* is part of the family *Fimoviridae* (order *Bunyavirales*) and includes plant viruses with a segmented, negative-stranded RNA genome. At least four genome segments are present in all members described to date [6]. We obtained the complete sequences of RNA 1 and RNA 2 of the novel virus by RT-PCR using species-specific primer pairs (see Supplemental Table S1) derived from HTS-based sequences. To amplify full-length genome segments of the putative RNA 3 (1.3 kb) and RNA 4 (1.4 kb) and to check for further genomic components, we performed RT-PCR using the terminal primer PDAP213 and velocity polymerase (Bioline) as described for other emaraviruses [7, 8]. PDAP213-primed RT-PCR amplified a putative fifth genome component, RNA 5, with a length of approximately 1 kb, from samples from three locations in Germany and Sweden. We cloned the full-length PCR products and sequenced two individual clones of RNA 3, four of RNA 4, and four of RNA 5 as described elsewhere [8]. BLASTx analysis (NCBI) confirmed that the cloned PCR products represent complete genome segments of the novel virus. Deduced aa sequences of the novel virus identified in oak showed 21-38% (RNA 3), 18-58% (RNA 4), and 18-41% (RNA 5) sequence identity to the corresponding protein sequences encoded by High Plains wheat mosaic virus (HPWMoV), raspberry leaf blotch virus (RLBV), ti ringspot-associated virus (TiRSaV) [9], jujube yellow mottle-associated virus (JYMaV) [10], and blue palo verde broom virus (PVBV) [11]. We determined the sequence termini of each RNA segment by rapid amplification of cDNA ends (RACE) as described [8], using specific primers complementary to the genomic and antisense strand, respectively, of each RNA (Table S1).

The putative functions of proteins encoded by RNA 1 to RNA 4 of this virus were deduced based on the presence of conserved domains related to those of other emaraviruses. The RNA-dependent RNA polymerase (RdRP) is encoded by RNA 1 (2317 aa, 271.7 kDa), while RNA 2 encodes the glycoprotein precursor (GPP, 651 aa, 74.2 kDa). The viral nucleocapsid protein (NC, 291 aa, 32.9 kDa) is encoded by RNA 3, and RNA 4 encodes the movement protein (MP, 365 aa, 41.7 kDa) (Fig. 1). We aligned amino acid sequences of the viral RdRP, GPP, NC, and MP of the novel virus identified in ringspot-diseased oak with sequences of homologous proteins of emaraviruses to establish their phylogeny. The novel virus clustered consistently with a group of emaraviruses comprising HPWMoV, RLBV, TiRSaV, JYMaV, and PVBV, regardless of the protein considered (Fig 2A. phylogenetic trees for GPP and MP not shown). Phylogenetic analysis and species demarcation criteria for emaraviruses (sequence divergence of over 25% for proteins encoded by viral RNA 1, RNA 2 and RNA 3 [12]) indicate that the virus represents a distinct species of the genus emaravirus. This conclusion is reinforced by the structure of the genome, which has (i) at least five RNA segments, each containing one ORF, (ii) conserved 13-nt sequences at the 5’ and 3’ termini of each RNA, and (iii) partial complementarity at the terminal ends of each genome segment. Based on the observed ringspot symptoms on leaves, we propose the name “common oak ringspot-associated emaravirus” (CORaV) for the novel virus.

**Fig. 1:**
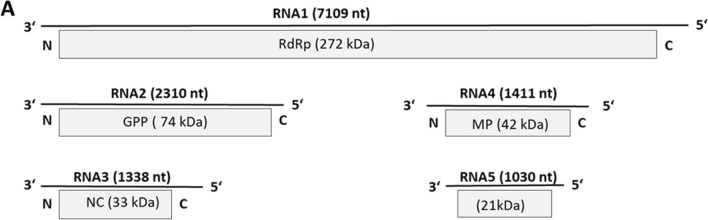
Genome organization of common oak ringspot-associated virus (CORaV). Genome segments are displayed as mRNA, with grey boxes representing the protein encoding region (ORF) for each RNA. The protein encoded by each RNA and its predicted molecular mass are given. RdRP, RNA-dependent RNA polymerase; GPP, glycoprotein precursor; NC, nucleocapsid; MP, movement protein

**Fig. 2:**
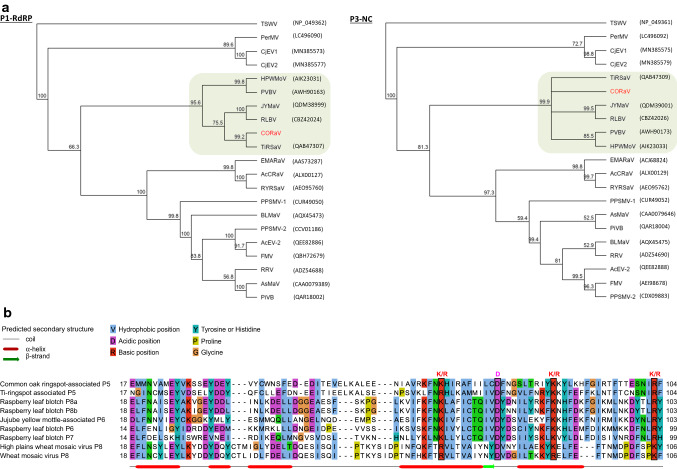
(A) Phylogenetic placement of common oak ringspot-associated virus (CORaV). We used MUSCLE to align amino acid sequences of the CORaV RdRP and NC with the corresponding sequences of other emaraviruses, including the assigned viruses actinia chlorotic ringspot-associated emaravirus (AcCRaV), blackberry leaf mottle-associated emaravirus (BLMaV), European mountain ash ringspot-associated emaravirus (EMARaV), fig mosaic emaravirus (FMV), High Plains wheat mosaic emaravirus (HPWMoV), pigeonpea sterility mosaic emaraviruses 1 and 2 (PPSMV-1 and PPSMV-2), pistacia emaravirus B (PiVB), raspberry leaf blotch emaravirus (RLBV), rose rosette emaravirus (RRV), and redbud yellow ringspot-associated emaravirus (RYRSaV) as well as the unassigned viruses actinidia virus 2 (AcEV-2), aspen mosaic-associated virus (AsMaV), Camellia japonica associated virus 1 and 2 (CjEV1 and CjEV2), jujube yellow mottle-associated virus (JYMaV), blue palo verde broom virus (PVBV), perilla mosaic virus (PerMV), and ti ringspot-associated virus (TiRSaV)). Phylogenetic trees were built using the neighbor-joining method in Geneious prime 2019.1.1 software. Accession numbers are indicated. Numbers on branches represent statistical support based on bootstrap analysis (1000 replicates). CORaV (red) and other emaraviruses that clustered into the same group are indicated by coloured boxes. Tomato spotted wilt virus (TSWV, genus *Orthotospovirus*) was used as an outgroup. (B) Alignment of a partial CORaV P5 sequence and its homologs. The accession numbers of the sequences used are as follows: QAB47311.1 (TiRSaV P5), AIK23039.1 (HPWMoV P8), AML03198.1 (HPWMoV P8), YP_009237280.1 (RLBV P8a), AKU41979.1 (RLBV P8b), QDM39004.1 (JYMaV P6), YP_009237279.1 (RLBV P7), AKU41976.1 (RLBV P6).

RNA 5 (1030 nt) of CORaV contains one major open reading frame, coding for a protein of 179 aa (P5, 21.1 kDa). We identified sequence similarity to the putative silencing suppressor P8 of HPWMoV [13] and to proteins encoded by other emaraviruses as described elsewhere [8], with sequence similarities ranging from 18% (RLBV P7) to 41% (TiRSaV P5). CORaV P5 is predicted to be mostly α-helical, and the alignment of its N-terminal moiety (Fig. 2B), which is the region best conserved across all homologs (aa 17-104 in CORaV P5), shows that one aspartate is strictly conserved in all species (D75 in CORaV P5), while three positions are semi-conserved, containing the basic amino acid arginine (R103) or lysine (K65 and K85). However, the function of P5 remains to be elucidated.

We correlated the detection of all five viral RNAs to the occurrence of the ringspot disease of oaks. Leaf material from healthy and ringspot-diseased oaks at over 25 locations in Germany, Sweden, and Norway was sampled from landscape, nursery-grown, and urban trees. In 191 out of 198 *Q. robur* samples, we confirmed the presence of viral RNAs by RT-PCR applying species-specific primers (Table S1) targeting all five genome segments. We observed chlorotic ringspots and spots on diseased trees as the most abundant symptoms (Supplemental Fig. S1, left side), and mottle was found only in a few samples. However, in seven trees displaying ringspot symptoms, we did not detect any of the tested CORaV genome segments. In leaf material collected from trees without any symptoms (n = 28), no viral genome segment could be detected. We obtained the same results for leaf samples from 25 trees that had never shown chlorotic ringspots but had either a regular chlorotic pattern or partial chlorosis of leaves (Supplemental Fig. S1, right side). The latter symptoms were therefore considered atypical of CORaV and not associated with the ringspot disease. We consider it likely that CORaV contains five genome segments and is associated with the ringspot disease in common oak. Since virus infections represent a significant predisposition for other diseases and stress, further studies are needed to fully understand the biology and epidemiology of CORaV as well as its impact on the health status of trees, a prerequisite for effective disease management.

## Supplementary Information

Below is the link to the electronic supplementary material.

**Supplemental Fig. S1** Symptoms observed on diseased *Q. robur* trees, with CORaV-associated chlorotic ringspots (left, red arrow) and atypical regular chlorotic patterns (right). **Supplemental Table S1** Primer pairs used for RT-PCR-based amplification of missing sequence information of the novel emaravirus identified in common oak including RACE and detection of viral RNA 1 - RNA5 (DOC 547 KB)
