# Supplementary material for: A novel emaravirus comprising five RNA segments is associated with ringspot disease in oak
Source: Arch Virol. 2021 Jan 18;166(3):987–90. doi: 10.1007/s00705-021-04955-w (PMC7884362; doi:10.1007/s00705-021-04955-w)
Supplement: Supplementary file 1 — Supplemental Fig. S1 Symptoms observed on diseased Q. robur trees, with CORaV-associated chlorotic ringspots (left, red arrow) and atypical regular chlorotic patterns (right). Supplemental Table S1 Primer pairs used for RT-PCR-based amplification of missing sequence information of the novel emaravirus identified in common oak including RACE and detection of viral RNA 1 - RNA5 (DOC 547 KB) [file 705_2021_4955_MOESM1_ESM.doc]

**Supplementary Materials**

**
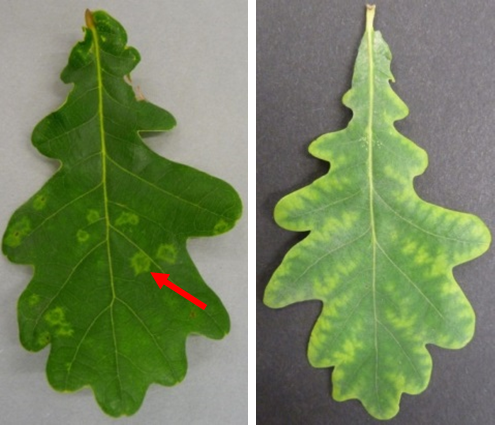
**

**Figure S1**. Symptoms observed on diseased *Q. robur* trees, with CORaV-associated chlorotic ringspots and confirmed virus infection (left, red arrow) in comparison with CORaV-atypical regular chlorotic patterns (right).

**Table S1.** Primer pairs used for RT-PCR-based amplification of missing sequence information of novel emaravirus identified in Common oak including RACE and detection of viral RNA 1 - RNA 5

| **Primer name** | **5´- sequence - 3´** | **genome region** | **reference** |
| --- | --- | --- | --- |
| **Full-length RT-PCR** | | | |
| **PDAP213** | GGCGACCCGCTCCGGTACCCTAGTAGTGAACTCC | all | Di Bello et al. 2015 |
| **Determination of complete RNA1 and RNA2 of novel emaravirus** | | | |
| **PDAP213** | See above | all | Di Bello et al. 2015 |
| **S36-742F** | GAAGTATATACTCTCGTTATCC | RNA1 | This study |
| **C329-1431F** | TCATTCAGAATCAATTAGGAATGG | RNA1 | This study |
| **C1727-100R** | GCCACAAAGTATCTAAGTGGAG | RNA1 | This study |
| **S134-95R** | GTATCAGTTATTGATAAGCTTGATATGG | RNA1 | This study |
| **S71-491F** | TAAAAGAACTGGTCCAACCAC | RNA1 | This study |
| **C1291-1029F** | CCATTCTTAAGTATGTGTGAACC | RNA2 | This study |
| **C1291-300R** | TATGATCATGATGGATTCACAGAG | RNA2 | This study |
| **RT-PCR based detection of RNA 1 – RNA 5** | | | |
| **C329rc-380F** | GAGACACCTAGCTTCTCAGT | RNA1 | This study |
| **C329rc-861R** | GGAGATCTTGATGTCATAATGTC | RNA1 | This study |
| **C1291-1029F** | CCATTCTTAAGTATGTGTGAA | RNA2 | Bandte et al 2020 |
| **C1291-1609R** | TATGATCATGATGGATTCACAGAG | RNA2 | Bandte et al. 2020 |
| **Emara-oak-NC-F2** | CAGAGCTATGGCTATCTGCA | RNA3 | Bandte et al. 2020 |
| **Emara-oak-NC-R2** | GTTGCTATCACTTCTGCAGG | RNA3 | Bandte et al. 2020 |
| **C755-514F** | CAAGCTCCTGAAGCTTATTCAACA | RNA4 | Bandte et al. 2020 |
| **C755-826R** | GAATCAATTGTTCAGATGAGCATG | RNA4 | Bandte et al. 2020 |
| **C105-69F** | ATCTTCTGAATATGATGAGTATG | RNA5 | This study |
| **C105-230R** | GAAAGAGAACCATTGAAGTCAC | RNA5 | This study |
| **C105-34F** | GAGGAGATGGTGAATGTTGC | RNA5 | This study |
| **C105-416R** | CCATCCTGGATCTGTAGTTTG | RNA5 | This study |
| **RACE primers** | | | |
| **S10-1655F** | ATATGCACTTAGAACTTTAC | RNA1 | This study |
| **S10-1830F** | CCATATCAAGAATTGATTAAC | RNA1 | This study |
| **C329-363R** | ATGTCTAGACATTTCAAGAA | RNA1 | This study |
| **C329-185R** | CAGATCTTCTAGAATTAACTA | RNA1 | This study |
| **C1291-1818F** | GCTTTCAATCTGATAAGTATG | RNA2 | This study |
| **C1291-1992F** | GAAGGTATAGAGTATGTATATG | RNA2 | This study |
| **C1291-224R** | CCATGAATGACTCATATACTT | RNA2 | This study |
| **C1291-389R** | GCATACAAGATATATGCCTCT | RNA2 | This study |
| **C881-703F** | GATGATATAATAAAATCAACTG | RNA3 | This study |
| **C881-839F** | ATGTTAGAATTGAAGACTTCA | RNA3 | This study |
| **C881-632R** | GCAGGAAAGTTATCAAATGT | RNA3 | This study |
| **C881-164R** | CCAGATAATGGAAAGAACTCA | RNA3 | This study |
| **C755-1053F** | ACATATTTCCTTAGAAATTCA | RNA4 | This study |
| **C755-1205F** | CCAATCAGAAATGTCATGATC | RNA4 | This study |
| **C755-418R** | ATTGAAAAGCTAGATAAGGCA | RNA4 | This study |
| **C755-254R** | TGACATTGTTAGCATGTACTAG | RNA4 | This study |
| **C105-590F** | CCTTTATCAGTTATTAAGGCA | RNA5 | This study |
| **C105-861F** | CACTTAAGAGTGATGATGGTT | RNA5 | This study |
| **C105-312R** | GTCACATAATATTATGAATGC | RNA5 | This study |
| **C105-163R** | CATATTCCATTGCAACATTCA | RNA5 | This study |

* virus specific sequence is underlined
